# Effect of Sarcoplasmic Protein Solutions Dried at Different Times and Rates on Water Migration in Lamb Myofibril In Vitro

**DOI:** 10.3390/foods13060930

**Published:** 2024-03-19

**Authors:** Weili Rao, Sijia Liu, Shiquan Kong, Zhenyu Wang, Zidan Shi, Jianming Cai

**Affiliations:** 1 College of Food Science and Technology, Hebei Agricultural University, Lekai South Avenue, Baoding 071000, China; iamraoweili@126.com (W.R.); liusijia131@126.com (S.L.); k2422023467@163.com (S.K.); 19931683486@163.com (Z.S.); cjm3414774618@163.com (J.C.); 2 Institute of Food Science and Technology, Chinese Academy of Agricultural Sciences, Integrated Laboratory of Processing Technology for Chinese Meat and Dish Products, Ministry of Agriculture and Rural Affairs, Beijing 100193, China

**Keywords:** sarcoplasmic protein, denaturation, myofibril, water migration, air-drying

## Abstract

To determine whether sarcoplasmic proteins affected water migration in myofibrils during air-drying, with protein denaturation as an indicator of sarcoplasmic protein changes, the extent of sarcoplasmic protein changes in lamb during air-drying was first studied. The results showed that sarcoplasmic protein’s thermal stability decreased and secondary structure changed, indicating sarcoplasmic protein denatured in lamb during air-drying (35 °C, 60% RH, 3 m/s wind speed). Subsequently, the effect of sarcoplasmic protein solutions, dried at different times and rates, on myofibril protein–water interaction was studied in vitro. Two sets of sarcoplasmic protein solutions were dried for 0, 3, 6, and 9 h in a drying oven, resulting in different degrees of change. These two sets with higher or lower drying rates were achieved by controlling the contact area between sarcoplasmic protein solution and air. These dried sarcoplasmic protein solutions were then mixed with extracted myofibril and incubated for 2 h. The results showed a significant increase in T_21_ relaxation time of the incubation system when sarcoplasmic protein solution was dried at 35 °C for 3 h. This indicated that myofibrillar protein–water interaction was weakened, facilitating water migration from the inside to the outside of myofibrils. The denaturation degree of sarcoplasmic proteins was slowed by a higher drying rate, thereby alleviating the increase in the amount of immobile water within myofibrils when dried for 6 h. In conclusion, the properties of sarcoplasmic proteins were influenced by both drying rate and time, thereby influencing the water migration within myofibrils during air-drying.

## 1. Introduction

Dried meat is a popular traditional commodity in China, with diverse processing techniques employed. In regions such as Inner Mongolia and Xinjiang of China, the traditional method for air-drying meat involves slicing raw meat (mainly beef and lamb) into strips, which are then hung in ventilated areas to reach the desired moisture content. Before eating, the air-dried beef or lamb is braised with a blend of spices. Nowadays, commercial enterprises have adapted and refined this traditional approach, utilizing raw meat for drying. However, owing to factors such as price fluctuations, seasonal variations in slaughter, and importation, raw meat utilized by meat production enterprises is predominantly frozen. Various drying methods are employed, including hot air-drying, ultrasonic drying, and freeze drying [1], with hot air-drying being currently widely used by Chinese enterprises. During the drying process, moisture migration from the meat to the drying medium affects the energy consumption, production cycle, and product quality of air-dried meat. Consequently, investigating the laws and mechanisms governing moisture migration during the drying process is crucial in the field of air-dried meat processing.

Since the 1920s, scholars globally have utilized molecular dynamics and thermodynamic approaches to investigate moisture migration phenomena within materials. They have proposed various driving forces for water transport, including gravity, concentration gradient, temperature gradient, and pressure gradient. Different mechanisms have been proposed for water migration based on mechanical and capillary flows [2,3,4,5,6]. However, a substantial portion of those researched in this field have focused on materials such as fruits, vegetables, grains, etc., resulting in a lack of research on moisture migration during the drying process of meat and meat products. Certain scholars have analyzed the moisture migration in the processing of meat products, such as refrigeration, curing, and roasting. These works have provided valuable insights for investigating moisture migration during the air-drying of meat. For instance, Radford [7] analyzed the potential mechanisms of moisture migration during the storage of raw meat, identifying Fick’s diffusion as the primary mode of moisture migration. Feyissa et al. [8] clarified that pressure was the predominant driving force during pork roasting, utilizing Darcy’s formula in conjunction with the mass–energy conservation law. Van der Sman [9] determined that the Flory–Rehner theory aligned more consistently with the moisture content and temperature distribution of meat during heating from 20 to 80 °C, accounting for protein denaturation simultaneously. In addition, Zheng et al. [10] suggested a relationship between the secondary structure of meat products and moisture migration. Therefore, in addition to investigating the mechanisms of moisture migration caused by physical factors, it is crucial to consider the influence of protein denaturation, protein degradation, and other biochemical reactions occurring during the drying process on moisture migration.

Ninety-nine percent of water in thawed meat is in a non-readily mobile state, primarily located within the myofibril. The interaction between myofibrillar proteins and water is directly related to the rate of water migration during the air-drying process. Within muscle tissue, the sarcoplasmic component is in direct contact with the myofibril. Several studies on pork have shown that the denaturation of sarcoplasmic proteins impacted meat quality parameters, including color and water-holding capacity [11,12]. In a study focused on PSE meat (pale, soft and exudative meat), Liu et al. [13] found that myofibrils within the sarcoplasm lacking denatured proteins exhibited the poorest water-holding capacity. Conversely, the water-holding capacity of myofibrillar proteins gradually increased with increased concentration of denatured sarcoplasmic proteins. Scholars hypothesized that the observed improvement in water-holding capacity was due to the aggregated and denatured sarcoplasmic proteins, effectively trapping water migrating from the myofibrils and increasing the water-holding capacity of the whole system. Rao et al. [14] observed a gradual decrease in the amplitude of the second peak in the DSC profile of air-dried meat, attributing the peak to the denaturation of myosin tails, collagen, and sarcoplasmic proteins. Further studies are needed to determine whether denaturation of the sarcoplasmic proteins occurred during air-drying. While some researchers have investigated the effect of sarcoplasmic protein content on myofibrils and meat’s water-holding capacity [15,16,17], the study of the degree of denaturation and its influence on the water-holding capacity of myofibrils during meat processing remains under-explored.

In this study, solutions of sarcoplasmic protein from air-dried meat with different moisture contents were extracted. These solutions were tested using DSC, circular dichroism, and SDS-PAGE to elucidate the degree of sarcoplasmic protein denaturation during the air-drying process. Subsequently, the interaction between protein and water of the incubation system of dried sarcoplasmic protein solutions and myofibrillar fibers was examined using low-field NMR in vitro experiments, thereby elucidating the form of water within the system. It is essential to note that, apart from protein denaturation of the sarcoplasmic protein solution, other physical and biochemical changes occurred during 35 °C air-drying. In this study, protein denaturation was utilized as an indicator to obtain different degrees of change in the sarcoplasmic protein solution. In our previous in vitro experiments, we found that the degree of denaturation of sarcoplasmic proteins was affected by the rate of dehydration. Higher water loss rates in the sarcoplasmic protein solution correlated with reduced protein precipitation and diminished color changes in the sarcoplasmic protein solution. It is possible that rapid water removal led to less water in the sarcoplasmic protein solution, and then, less water content affected the denaturation of sarcoplasmic proteins. Consistent with our findings, other researchers have observed that higher moisture content accelerated denaturation [18,19]. Therefore, the effects of different water loss rates (also called different drying rates) on sarcoplasmic protein denaturation and on protein–water interaction within myofibrils was further investigated. These interactions ultimately influence the water migration within myofibrils during air-drying. This work contributes fundamental data and insights for understanding the mechanism of water migration during traditional air-dried meat processing.

## 2. Materials and Methods

### 2.1. Experiment 1: Confirmation of the Degree of Denaturation of Sarcoplasmic Proteins in Air-Dried Lamb Meat

#### 2.1.1. Sample Collection and Air-Drying of Lamb

Nine 6-month-old small-tailed Han sheep rams, housed together in the same pen, were obtained from a commercial slaughterhouse (Caoyuanhongbao Co., Ltd., Inner Mongolia, Bayannur, China). Following halal slaughter, the carcasses were stored for 48 h at 4 °C. The semimembranosus muscles from the two hind legs were sectioned, wrapped in tin foil, and placed within self-sealing bags (2300 mL O_2_/(m^2^·24 h) at 0 °C). Subsequently, the meat was frozen in dry ice (−78 °C) and transported back to the laboratory within approximately 20 h. Upon arrival at the laboratory, the meat was stored at −20 °C for one month.

Before drying, the meat was thawed at −0.5 °C for 24 h in a refrigerator. Subsequently, the meat was sliced into cuboids measuring 1.5 × 1.5 × 3 cm^3^, with the long axis of the cuboids aligned parallel to the direction of the muscle fibers. Following the cutting process, the meat cuboids were placed in a refrigerator set at 4 °C. Once the center temperature of the meat reached 4 °C, it was removed for drying. It should be noted that approximately 10% of the juice from the meat cuboids was lost during the thawing and cutting stages. All cuboids were suspended on homemade hooks, ensuring the muscle fibers’ orientation was perpendicular to the ground. The cuboids were then placed in an air-drying oven (D/GDWJB-100L, Shanghai Dianhe Testing Instrument Factory, Shanghai, China) equipped with an automatic weighing system. Air-drying was conducted under controlled conditions typically used by certain Chinese meat drying enterprises: a temperature of 35 °C, a relative humidity of 60%, and a wind speed of 3 m·s^−1^. Twenty pieces of meat were hung in the automatic weighing system, and the weight of the strips (initial moisture content of 74.00% ± 1.46%) was recorded at 5 min intervals. Samples were randomly removed when their moisture content dropped to 70%, 65%, 60%, 55%, 50%, and 45%.

#### 2.1.2. Extraction of Sarcoplasmic Proteins

Air-dried meat samples with varying moisture content were immersed (*w*/*v* = 1:4) in a precooled extracting buffer (0.1 M NaCl, 2 mM MgCl_2_, 1 mM EGTA, pH = 7.0). The mixture was homogenized at high speed and centrifuged at 10,000× *g* at 4 °C for 15 min. The resulting supernatant was used as a sarcoplasmic protein solution.

#### 2.1.3. Myofibrillar Protein Extraction

Myofibrillar proteins were extracted using the method described by Park et al. [20]. Air-dried meat samples with varying moisture content were immersed (*w*/*v* = 1:4) in a precooled extracting buffer (0.1 M NaCl, 2 mM MgCl_2_, 1 mM EGTA, pH = 7.0). Following high-speed homogenization for 5 s with a 10 s pause, repeated four times, the mixture was centrifuged at 2000× *g*, at 4 °C for 15 min. The resulting precipitate was placed in the same buffer, subjected to homogenization, and underwent two consecutive centrifugation steps. Subsequently, the material was homogenized with four times the volume of 0.1 M NaCl solution, followed by two additional centrifugation steps. Before the final centrifugation, the solution was filtered through a single layer of gauze. The obtained myofibrillar protein was dissolved in a 5% SDS solution, homogenized for 30 s, and stored at −80 °C. Before electrophoresis, the protein concentration was determined, with 20 μg of protein loaded into each well.

#### 2.1.4. Differential Scanning Calorimeter (DSC) of Sarcoplasmic Protein

Three air-dried meat cuboids of varying moisture content were used to separate the interior meat or the surface hard crust. Sarcoplasmic protein solutions were extracted separately from the interior and the surface hard crust of every air-dried meat cuboid. The protein concentration of the sarcoplasmic protein solution was adjusted to 44 mg/mL. Subsequently, the thermodynamic properties of these sarcoplasmic protein solutions upon heating were determined to assess the denaturation degree using DSC (Q200, TA Instruments Ltd., Delaware, NC, USA), which was calibrated with the thermal characteristics of indium before testing. Sarcoplasmic protein samples (15–20 mg) were heated at a rate of 10 °C/min, ranging from 20 °C to 90 °C. A Q200 empty pan served as the blank. The thermal denaturation temperature (T_max_) and the enthalpy of absorption (J/g) of the proteins were analyzed using the software supplied with the instrument. The DSC experiment was repeated three times for the interior and the surface hard crust of every meat cuboid.

#### 2.1.5. Circular Dichroism (CD) Spectral

Three air-dried meat cuboids of each moisture content were used for extracting sarcoplasmic proteins separately. The sarcoplasmic protein solution underwent spectral recording within the wavelength range of 190 to 250 nm using a circular dichroism spectropolarimeter (MOS-500, BioLogic, Seyssinet-Pariset, AuRA, France) at 25 °C. The sample concentrations were 500 μg/mL for the chemical analysis, and the circular dichroic cuvette used was 0.1 cm thick.

#### 2.1.6. SDS-PAGE

Three air-dried meat cuboids of each moisture content were used for extracting myofibrillar proteins separately. SDS-PAGE was performed using a 12% separation gel. Following electrophoresis, the gels underwent three consecutive 10 min washes with ultrapure water. Subsequently, the gels were soaked in SYPRO Ruby (S12000, Invitrogen, Carlsbad, CA, USA) staining solution for 12 h, followed by decolorization in decolorization solution (10% ethanol, 7% acetic acid) for 30 min under light protection, repeated three times. Finally, the gels were washed with ultrapure water for 10 min, repeated three times.

### 2.2. Experiment 2: Confirmation of Denaturation of Sarcoplasmic Proteins in In Vitro Model

#### 2.2.1. Determination of Method of Sarcoplasmic Proteins Extraction

The collection process for lamb meat was identical to the procedure described in Section 2.1.1. After collection, the meat was stored in a refrigerator set at −20 °C for one month. Subsequently, the meat was thawed at 4 °C for 24 h. It was then divided into three parts for sarcoplasmic protein extraction using three different methods.

One portion of the thawed meat samples was immersed (*w*/*v* = 1:4) in precooled extracting buffer (0.1 M NaCl, 2 mM MgCl_2_, 1 mM EGTA, pH = 7.0). After high-speed homogenization using a dispersion machine (T10, IKA, Staufen, Germany), the resulting homogenate was centrifuged at 10,000× *g*, at 4 °C for 15 min. The supernatant was labeled as buffer-extracted sarcoplasmic proteins (BESP). A second portion of the thawed meat samples was cut into 0.5 cm strips and centrifuged at 10,000× *g* for 20 min. The resulting supernatant was named the centrifugal extraction sarcoplasmic protein solution (CESP). The remaining thawed samples had exuded juice collected during thawing, which was used as a sarcoplasmic protein solution (JESP). The protein concentrations of the sarcoplasmic protein solutions extracted through these three methods were determined using a BCA kit (Thermo Fisher Scientific Inc., Waltham, MA, USA), and the protein concentration was adjusted to 2 μg/mL. Subsequently, SDS-PAGE was performed using a 12% separation gel to compare the three extraction methods and select the most appropriate one for subsequent experiments.

#### 2.2.2. Confirmation of Denaturation of Sarcoplasmic Proteins In Vitro Model

Five tubes containing 15 mL of CESP obtained from thawed meat were placed in an air-drying oven (35 °C, RH 60%, wind speed 3 m/s). One tube was removed from the oven every hour and stored at 4 °C in a refrigerator. Subsequently, the pH and protein concentration of the centrifuged supernatant from each tube were measured.

### 2.3. Experiment 3: Effect of Drying Time and Drying Rate of CESP on Denaturation of Sarcoplasmic Proteins and on Myofibril–Water Interactions In Vitro Experiments

#### 2.3.1. The Effect of Drying Time and Drying Rate of CESP on Sarcoplasmic Protein Denaturation

Lamb meat was collected using the same methodology as detailed in Section 2.1.1. The meat was then stored at −20 °C for one month. Subsequently, CESP was extracted as described in Section 2.2.1. To obtain distinct sarcoplasmic protein suspension, 10 mL portion of CESP was introduced into either a 20 mL sample bottle (with a 3.14 cm^2^ contact area with air, and the lower drying rate was 0.00015 min^−1^) or a 100 mL beaker (with a 26.5 cm^2^ contact area with air, and the higher drying rate was 0.0018 min^−1^). The drying rate was calculated based on the weight loss per minute and per gram of wet weight of CESP. These containers were then placed inside a drying oven for 0, 3, 6, and 9 h. Due to the different contact areas with air, the water evaporation rates differed. Samples were taken out and adjusted to 10 mL. The protein concentration of the supernatant obtained after centrifugation was subsequently determined.

#### 2.3.2. Incubation

The sarcoplasmic protein suspension obtained, as described in Section 2.3.1, was thoroughly mixed with myofibril and incubated for 2 h. The volume of the sarcoplasmic solution and the weight of myofibril were calculated based on the protein concentration of both components. In the in vitro incubation experiments, the ratio of sarcoplasmic protein and myofibril protein was maintained at 1:1.5.

#### 2.3.3. Low-Field NMR of the Incubation Mixture of Sarcoplasmic Protein and Myofibril

The low-field NMR parameters for hydrogen protons used in this study followed the method described by Rao et al. [14]. The specific parameters were as follows: a magnetic field strength of 0.5 T, proton resonance frequency of 23 MHz (SF = 23 MHz), Q1 = 286.7813 kHz, P90 = 17 µs, P180 = 35 µs, TD = 54,996, TR = 3000 ms, NS = 4, and Echo Count = 200 (NMR-2011, Niumag Electric Corporation, Shanghai, China). The CPMG exponential decay curves were obtained, and the T-values were extracted using the MultiExpInv Analysis software A.20230410.68923 provided with the instrument. The final results were normalized to mass.

Sarcoplasmic protein with varying degrees of denaturation, obtained from the two samples in Section 2.3.1, underwent co-incubation with myofibril for 2 h. The mixture was placed in an ice bath for 30 min and subsequently analyzed using low-field NMR.

### 2.4. Statistical Analysis

The results in this study were expressed as mean ± standard deviation. For both Experiment 1 and Experiment 2, the data were evaluated through one-way ANOVA using SPSS v.22.0 (IBM Corporation Inc., Armonk, NY, USA). Significance analysis was performed using the LSD method (*p* < 0.05). For Experiment 3, two-way ANOVA, with fixed factors of drying time (3, 6, and 9 h) and drying rate, was used to analyze the effect of drying time, drying rate, and the interaction between drying time and drying rate on relaxation time, the area, and the ratio of T_2b_, T_21_, and T_22_ of mixtures of myofibrillar and sarcoplasmic proteins using SPSS. Post hoc tests for ANOVA included the general linear model and LSD, with differences considered statistically significant at *p*-values < 0.05.

## 3. Results and Discussion

### 3.1. Experiment 1: Confirmation of the Degree of Sarcoplasmic Protein Denaturation in Air-Dried Meat

#### 3.1.1. Sarcoplasmic Protein Denaturation in Air-Dried Lamb Determined by DSC

The results presented in Table 1 show that sarcoplasmic proteins extracted from raw lamb meat (with a moisture content of 74%) had a single thermal absorption peak with a peak apex temperature of 67.51 °C. Sarcoplasmic proteins extracted from air-dried meat, even with a minimal dehydration level of 4%, displayed a significantly lower peak apex temperature than that of the sarcoplasmic protein derived from raw lamb meat. The peak apex temperature of the sarcoplasmic proteins from raw lamb meat closely corresponded to the maximum of the second absorption peak temperature range observed in the DSC profile of raw meat, as reported by Zhang et al. [21]. As described in the literature, the denaturation of sarcoplasmic proteins can shift the heat-absorbing peak apex in DSC [22,23,24]. The lower peak apex temperature of sarcoplasmic proteins extracted from air-dried lamb meat indicated that the air-drying process led to a deterioration in the stability of the sarcoplasmic protein structure beyond the primary level [25], leading to denaturation.

#### 3.1.2. Sarcoplasmic Protein Denaturation in Air-Dried Lamb Determined by CD

**Figure 1:**
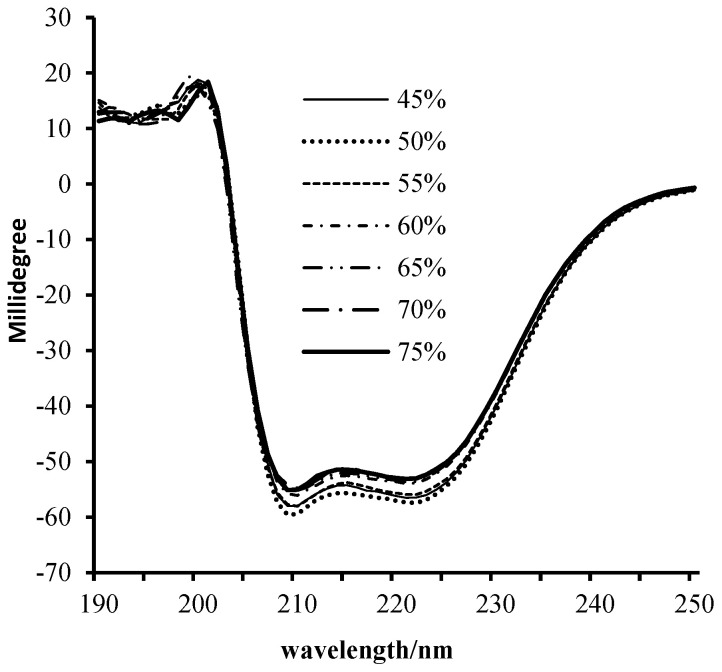
Circular dichroism spectrum of the extracted sarcoplasmic protein fraction from air-dried lamb with different moisture content. Sarcoplasmic proteins were extracted separately from three air-dried meat cuboids for each moisture content.

Figure 1 indicated there were no shifts in peak positions, and no missing peaks were observed in the CD chromatograms of sarcoplasmic protein solutions from air-dried meat with different moisture contents. The ratios of the peaks’ absorptivity minimum value are presented in Table 2. The ratios of 210/199 and 222/200 exhibited a significant increase followed by a decrease, indicating an alteration in the secondary structure of sarcoplasmic protein within the air-dried meat samples. In the CD results of sarcoplasmic proteins, the absorption peak at 200 nm was attributed to the β-folding of the protein, while the absorption peaks at 208 nm and 222 nm were associated with the a-helix. The ratio of β-folding to α-helix affects the structural stability of the protein [26]. Analyzing the absorbance ratios of these peaks allows for the determination of any changes in the secondary structure composition of the sample proteins. The results presented in Figure 1 indicate significant differences in the structures of sarcoplasmic proteins among air-dried meat samples with varying moisture contents.

#### 3.1.3. SDS-PAGE Analysis of Myofibrillar Proteins from Air-Dried Meat with Different Moisture Contents

**Figure 2:**
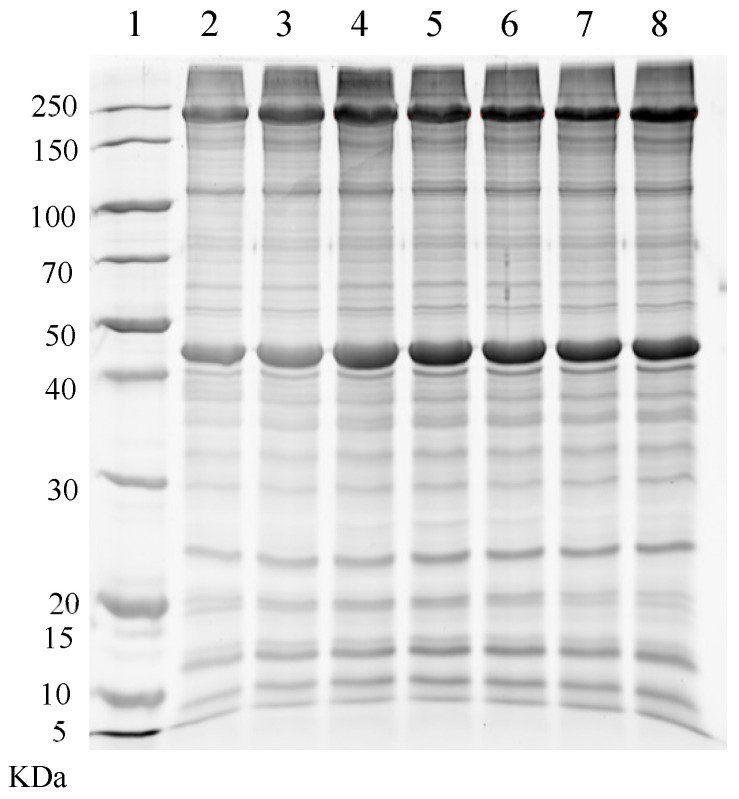
SDS-PAGE of myofibril protein from air-dried lamb meat with different moisture content, with 1 representing the marker, 2 corresponding to thawed meat with 74% moisture content, and 3, 4, 5, 6, 7, and 8 representing dried meat with 70%, 65%, 60%, 55%, 50%, and 45% moisture content, respectively. Sarcoplasmic proteins were extracted separately from three air-dried meat cuboids for each moisture content.

Figure 2 does not reveal any new or increased grey value bands, suggesting a difference between the in situ and in vitro models. If sarcoplasmic proteins had precipitated during the air-drying process, they would have co-precipitated with myofibrils and become visible in myofibrillar protein SDS-PAGE plots. Certain factors, such as reduced moisture levels during the air-drying process of the air-dried meat, likely counteracted any decrease in the solubility of myofibrillar protein. Moreover, the degeneration of sarcoplasmic proteins could not be detected through the SDS-PAGE method, given its semi-quantitative nature.

### 3.2. Experiment 2: Confirmation of the Denaturation of Sarcoplasmic Proteins in an In Vitro Model

**Figure 3:**
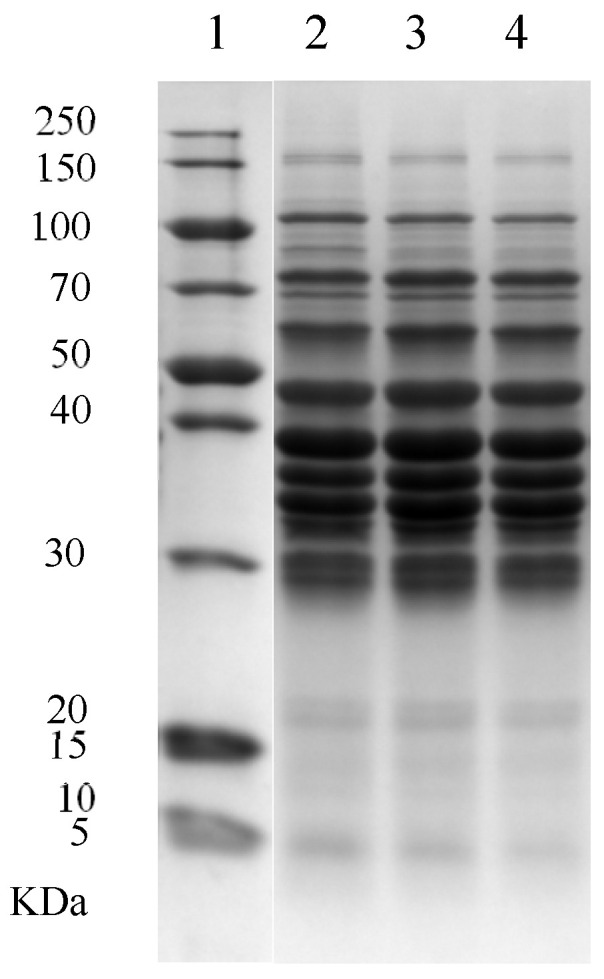
SDS-PAGE results of 1: marker, 2: sarcoplasmic protein extracted by buffer (BESP), 3: sarcoplasmic protein in juice exuded from thawed meat (JESP), and 4: sarcoplasmic protein in centrifugal extraction juice (CESP) of thawed meat.

A comparison of protein profiles between BESP, CESP, and JESP was performed using SDS-PAGE to assess the suitability of the juice exuded during the thawing process as a sarcoplasmic protein solution. As illustrated in Figure 3, the protein species and content of BESP, CESP, and JESP were generally similar, with subtle differences observed, especially in the protein bands within the 70–100 kDa range. The gray value of this band in JESP and CESP was lower than that in BESP, potentially due to the variations in conditions (such as ionic strength and pH) between the buffer used for sarcoplasmic protein extraction and the environment during thawing. The pH of the thawed meat was 5.51 ± 0.19, the pH of JESP was 5.51 ± 0.09, and the pH of CESP was 5.50 ± 0.12, with no significant difference observed among the three (*p* > 0.05). However, CESP had a higher protein concentration and closely resembled the physicochemical index of sarcoplasmic proteins in raw meat, as no additional chemicals from the extraction buffer were added. Therefore, CESP was selected as the sarcoplasmic protein solution for subsequent experiments (Experiments 2 and 3).

**Figure 4:**
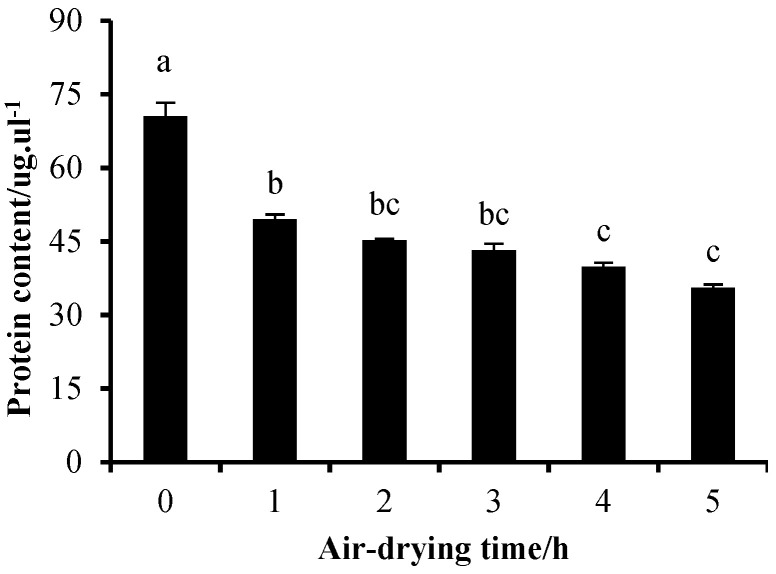
Change in sarcoplasmic protein solubility with different treatment time. The sarcoplasmic protein solution was placed in the air-drying machine at 35 °C for 5 h. All data were presented as mean ± standard deviation. Means with different lowercase letters were significantly different (*p* < 0.05).

**Figure 5:**
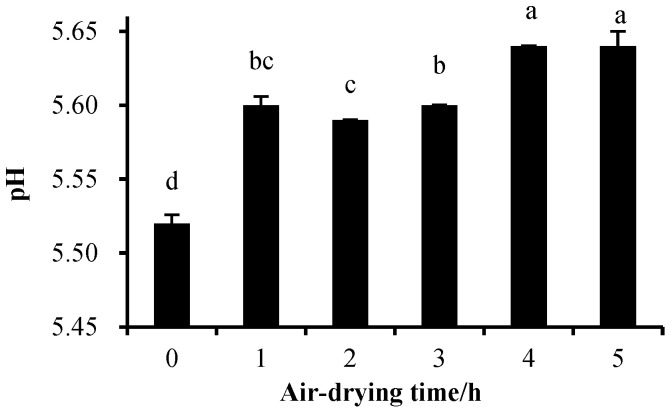
Change in pH of sarcoplasmic protein solution treated over different times. All data were presented as means ± standard deviation. Means with different lowercase letters were significantly different (*p* < 0.05).

The sarcoplasmic protein solutions were placed in an air-drying oven (35 °C, RH 60%, wind speed 3 m/s). As illustrated in Figure 4, the soluble protein content in the supernatant after centrifugation of the sarcoplasmic protein solution was significantly decreased (*p* < 0.05) after 1 h of treatment in the air-drying chamber. This decrease indicated that sarcoplasmic proteins were denatured, and their solubility decreased. Furthermore, the pH of the sarcoplasmic protein suspensions showed a significant increase with longer treatment times (Figure 5). The denaturation of sarcoplasmic proteins observed in this study can be attributed to the combined effect of temperature and pH, as supported by previous findings. Studies have indicated that changes in protein structure and decreased solubility occurred at 40 °C, with highly denatured proteins typically associated with lower pH values [27,28,29]. Joo et al. reported that differences in pH contributed to variations in sarcoplasmic protein solubility [12]. Yang et al. observed more pronounced denaturation of sarcoplasmic proteins at pH 5.5 and 40 °C compared to pH 6.2 and 40 °C in their study on pork [30]. Additionally, Fischer et al. discovered a reduction in the activity and solubility of sarcoplasmic protein phosphorylase within the temperature range of 36–40 °C and a pH range of 5.3–5.8 in rigor mortis muscle [31].

At relatively low pH and high temperatures, sarcoplasmic proteins may denature and precipitate on myofibrils, incorporating various glycolytic enzymes within the precipitated sarcoplasmic proteins [32]. Additionally, proteins, such as phosphorylase and creatine kinase, adhered to myofibrils and became visible in myofibrillar protein electrophoresis plots [33,34,35,36,37]. The potential precipitation of sarcoplasmic proteins during air-drying could lead to their co-extraction with myofibrils as precipitates, which would then be observed in myofibrillar protein SDS-PAGE plots. Although Figure 2 does not reveal any new or increased grey value bands, the results indicated a difference between the in situ and in vitro models. It is likely that certain factors, such as decreased moisture content during the air-drying of meat, mitigated the decrease in the solubility of sarcoplasmic protein.

### 3.3. Experiment 3: Effect of Drying Time and Drying Rate of CESP on the Denaturation of Sarcoplasmic Proteins and on Myofibril Protein–Water Interactions In Vitro Experiments

The solubility of sarcoplasmic proteins significantly decreased with increasing air-drying time (Table 3). However, the soluble protein content of sarcoplasmic proteins dried under a higher drying rate was significantly higher than that observed at a lower drying rate. The degree of denaturation of a protein affects its solubility [38]. The higher drying rate facilitated faster evaporation of moisture into the drying medium, consequently leading to a reduction in moisture content. Denatured sarcoplasmic protein can alter the water-holding properties of the mixture of myofibril fibers and sarcoplasm. In the in vitro model study conducted by Liu et al. [13], it was observed that myofibrils retained the least amount of water in the incubation system when sarcoplasmic proteins were absent. The presence of denatured sarcoplasmic proteins correlated with a higher water-holding capacity of the myofibrils. It was hypothesized that denatured sarcoplasmic proteins caused the shrinkage of the myofibril lattice, promoting water expulsion from the myofibrils. However, the denatured sarcoplasmic proteins could lock up this water, increasing the water retention in the incubation system. Nevertheless, some scholars proposed a different explanation, suggesting that water molecules are bound to the myofibril network through hydrogen bonding and that the decrease in the solubility of sarcoplasmic proteins led to increased protein–protein interactions, forming a more tightly packed network and binding more water [39].

The findings of this experiment show that sarcoplasmic proteins undergo denaturation with increasing air-drying time in an in vitro model. However, faster moisture loss rates slow down the degree of denaturation of sarcoplasmic proteins, leading to an increase in solubility. This finding also explains why the sarcoplasmic protein component did not appear in the myofibrillar protein SDS-PAGE of air-dried meat in Section 3.1.3. Specifically, the moisture content of the air-dried meat gradually decreased during the air-drying process, inhibiting the denaturation of sarcoplasmic proteins and resulting in higher protein solubility. As a result, during the extraction of myofibrillar proteins, there was no sedimentation of sarcoplasmic proteins along with the myofibrillar protein, which would otherwise be evident in the myofibrillar protein SDS-PAGE profile.

In this experiment, sarcoplasmic proteins exhibited varying degrees of denaturation at different drying rates. These denatured sarcoplasmic proteins were then co-incubated with myofibrillar proteins, and the results obtained from low-field NMR are summarized in Table 4. For fresh meat, the first peak (T_2b_) ratio was approximately 1%, with a relaxation time ranging from 1 to 5 ms. The ratio of the second peak (T_2b_) was more than 0.85%, with a relaxation time ranging from 11 to 17 ms. The ratio of the third peak (T_21_) was more than 97%, with a relaxation time ranging from 190 to 220 ms. In contrast, thawed meat T_2_ had three peaks distributed in the relaxation time of 1 to 1000 ms, which were 7 ms (T_2b_), 52 ms (T_21_), and 606 ms (T_22_) [14]. The appearance time of the third peak in this experiment was between the second and third peaks of thawed meat [14]. This result aligned with the findings of Berteam et al. [40], indicating that the peak appearance time of the peak with the largest area in the T_2_ relaxation of actin was around 100 ms. After actin formed a gel, the peak appearance time significantly decreased, indicating that actin required the establishment of a mesh-like structure to convert water in the system into immobile water that resists easy flow [26]. Low-field NMR measured the T_2_ relaxation time, where a larger relaxation time suggested water that is less tightly bound to proteins [10]. Therefore, the increase in the peak apex time of the third peak in this experiment may be attributed to the disruption of the original reticular structure of thick filaments and thin filaments in myofibrils during the extraction process. Additionally, the capillaries in the muscle nodes might have been destroyed.

Table 4 indicated that the interaction between air-drying time and drying rate did not significantly affect the relaxation time, peak area, and the ratio of Peaks 1, 2, and 3 in the mixture of myofibril and sarcoplasmic proteins solution, except for the peak area of Peak 1 (*p* = 0.044). However, the drying rate had a significant impact on the relaxation time (*p* < 0.001), peak area (*p* < 0.001), and ratio (*p* = 0.012) of Peak 1 in the mixture of sarcoplasmic proteins and myofibril. Except for the peak area of Peak 2 (*p* = 0.045), the drying rate did not significantly affect the relaxation time and ratio of Peak 2 in the mixture of myofibril and sarcoplasmic proteins solution. In addition, the drying rate significantly affected the peak area and ratio of Peak 3 (*p* < 0.05), but not its relaxation time. The air-drying time significantly affected the relaxation time, peak area, and ratio of Peaks 1, 2, and 3 in the mixture of myofibril and sarcoplasmic proteins solution (*p* < 0.05).

In our findings, two peaks appeared between the 1 ms and 20 ms relaxation times. The T_2b_ relaxation time (Peak 1 and Peak 2) showed significant changes with increasing air-drying time. Furthermore, the ratio of Peak 1 decreased with increasing air-drying time. The initial peak (T_2b_) was identified as water molecules that adhered to the surface of protein molecules, referred to as bound water. Depending on the degree of binding to the protein, bound water can be categorized as compound water, vicinal water, or multilayer water, with differences in the degree of binding leading to differences in relaxation times [41]. The change in relaxation time of T_2b_ indicated that the binding of water and protein surfaces in the system (sarcoplasmic proteins solution mixed with myofibrillar) was altered. Possible reasons for the difference in the degree of binding include alteration of the degree of water binding by protein denaturation and the shift of some strongly bound water to less strongly bound water [42]. The hot air-drying process in this experiment denatured the proteins in the sarcoplasm, weakening their binding to water and reducing the interaction forces between proteins and water [43]. The decrease in the ratio of Peak 1 with increasing air-drying time indicated a shift in water contributed to Peak 1 towards the water represented by Peak 2 or Peak 3. Additionally, the T_2b_ (Peak 1) relaxation time for the system of sarcoplasmic proteins mixed with myofibrillar proteins was shortened in beakers with a faster drying rate. This influence suggested that a faster drying rate may attenuate the degree of denaturation of the proteins during air-drying, allowing the interaction forces between the proteins and water to change more slowly.

In the present study, a significant increase was observed in the T_21_ relaxation time of the incubation system comprising sarcoplasmic proteins solution (treated at 35 °C for 3 h) mixed with myofibrils. While the lower air-drying rate did not significantly affect the T_21_ relaxation time compared to the higher air-drying rate, it did affect the ratio of T_21_. The ratio of T_21_ at higher drying rate was lower than the ratio of T_21_ at a lower drying rate. The peak (T_21_) was considered to be the immobilized water within the myofibril. The observed increase in the relaxation time of T_21_ after 3 h of air-drying of the sarcoplasmic protein solution demonstrated that the myofibrillar protein–water interaction was reduced due to changes in the sarcoplasmic protein solution. This change facilitated water migration from the interior to the exterior of myofibrils during air-drying. If so, an increase in drying rate would be observed because of the change in the sarcoplasmic proteins during air-drying. But the reality was that the drying rate of lamb was always decreasing during air-drying [14]. That is because the drying rate is also related to the nature of the drying layer on the surface of the lamb during air-drying. As the air-drying time increased, surface water was rapidly removed, resulting in surface shrinkage and a reduction or elimination of pores. Consequently, under the water gradient, water within the meat must be separated from the inner myofibrils first, and then migrated to the surface and combined with the myofibrils’ protein and other proteins on the surface of the dry layer, and then evaporated into the air. In summary, the speed at which myofibrils dissociate and reabsorb water collectively determines the rate of water drying. A lower drying rate induced a greater degree of denaturation of sarcoplasmic proteins. These findings indicate that more denatured sarcoplasmic proteins might increase the amount of immobile water in the myofibrillar network.

As described in the literature, in addition to protein denaturation, sarcoplasmic protein solutions also undergo changes, such as modification and degradation, or participate in biochemical reactions such as glycolysis and oxidative stress during storage [44]. However, there is a lack of research on whether these changes occur during air-drying. In this study, it was observed that sarcoplasmic protein solutions dried at different drying times and rates influenced the interaction between myofibril proteins and water during in vitro experiments. The relationship between sarcoplasmic protein denaturation and water migration in myofibrils was also specifically focused on and discussed. Nevertheless, further investigations are required to explore the effects of other physical and biochemical changes in sarcoplasmic proteins on water migration in dried meat.

## 4. Conclusions

DSC and circular dichroism studies revealed a decrease in the thermal stability and changes in the secondary structure of sarcoplasmic proteins, indicating denaturation during the 35 °C air-drying process of lamb meat. The results of the in vitro experiment indicated that the denaturation degree of sarcoplasmic proteins during air-drying was caused by a combination of drying rate and drying time. Furthermore, the in vitro experiments showed that the denaturation degree of sarcoplasmic proteins was slowed down by higher drying rate. The myofibrillar protein–water interaction was affected by sarcoplasmic protein solution dried at different times and rates. The myofibrillar protein–water interaction was weakened when sarcoplasmic protein solution was dried at 35 °C for 3 h, resulting in easier water migration from the interior to the exterior of myofibrils. A higher drying rate alleviated the increase in the amount of immobile water within myofibrils when sarcoplasmic proteins solution was treated at 35 °C for 6 h. This study focused on the relationship between sarcoplasmic protein denaturation and water migration in myofibrils. However, further investigations are needed to understand the effects of other physical and biochemical changes in sarcoplasmic proteins on water migration during air-drying.

## Figures and Tables

**Table 1:** T_apex_ and ΔH of heat absorption of sarcoplasmic protein extracted from the interior or surface crust of air-dried lamb with different moisture content, as determined by DSC.

Moisture Content (%)	T_apex_ (°C)		ΔH (J·g^−1^)	
	Interior	Surface Crust	Interior	Surface Crust
74	67.51 ± 0.88 ^a^	67.51 ± 0.88 ^a^	0.06 ± 0.02	0.06 ± 0.02
70	63.21 ± 0.49 ^b^	61.73 ± 1.54 ^b^	0.05 ± 0.01	0.11 ± 0.02
65	64.34 ± 1.46 ^b^	61.74 ± 0.88 ^b^	0.06 ± 0.01	0.10 ± 0.02
60	62.96 ± 1.03 ^b^	61.76 ± 1.23 ^b^	0.07 ± 0.02	0.09 ± 0.01
55	62.82 ± 1.24 ^b^	61.56 ± 0.51 ^b^	0.08 ± 0.00	0.10 ± 0.01
50	63.72 ± 0.04 ^b^	61.12 ± 2.36 ^b^	0.06 ± 0.02	0.11 ± 0.03
45	62.90 ± 0.17 ^b^	62.46 ± 0.16 ^b^	0.10 ± 0.01	0.07 ± 0.00

In each column, the presence of ‘^a^’ and ‘^b^’ indicates significant differences (*p* < 0.05). There was no significant difference in T_apex_ between the surface and the interior of the air-dried meat, and the data were not labeled with letters. Three air-dried meat cuboids with varying moisture content were used to separate the interior from the surface hard crust.

**Table 2:** Ratios of the peaks’ absorptivity minimum value in the circular dichroism spectrum of the extracted sarcoplasmic protein fraction from air-dried lamb with different moisture contents.

Moisture Content (%)	210/199	222/199	222/200
74	−4.03 ± 0.62 ^b^	−3.36 ± 0.18 ^a^	−3.87 ± 0.61 ^b^
70	−3.21 ± 0.21 ^ab^	−3.18 ± 0.21 ^a^	−3.09 ± 0.20 ^ab^
65	−2.98 ± 0.66 ^a^	−3.13 ± 0.76 ^a^	−2.88 ± 0.64 ^a^
60	−3.56 ± 0.42 ^ab^	−3.13 ± 0.31 ^a^	−3.41 ± 0.39 ^ab^
55	−3.62 ± 0.53 ^ab^	−3.31 ± 0.61 ^a^	−3.48 ± 0.53 ^ab^
50	−4.12 ± 0.45 ^b^	−3.77 ± 0.23 ^a^	−3.96 ± 0.43 ^b^
45	−3.41 ± 0.41 ^ab^	−3.17 ± 0.26 ^a^	−3.31 ± 0.43 ^ab^
*p*-Value	0.032	0.251	0.012

^a,b^ Means in the same column with different letters indicate significant difference (*p* < 0.05). Sarcoplasmic proteins were extracted separately from three air-dried meat cuboids for each moisture content.

**Table 3:** The effect of drying time and drying rate on the solubility of sarcoplasmic proteins (μg.μL^−1^).

Drying Time (h)	Drying Rate (min^−1^)		*p*-Value		
	0.0018	0.00015	Drying Time	Drying Rate	Drying Time × Drying Rate
0	95.28 ± 0.64 ^a^	95.28 ± 0.64 ^a^	0.000	0.000	0.004
3	88.92 ± 0.70 ^b^	84.27 ± 1.24 ^c^			
6	84.82 ± 1.40 ^c^	80.07 ± 0.14 ^d^			
9	78.22 ± 1.73 ^e^	75.35 ± 1.05 ^f^			

Different lowercase letters indicate significant differences (*p* < 0.05). Different moisture drying rates were controlled by adjusting the contact area between the sarcoplasmic protein solution and dry air. The larger the contact area, the greater the water removal rate and the less water in the sarcoplasms.

**Table 4:** Effect of drying rate and drying time on relaxation time, area, and the ratios of T_2b_, T_21_, and T_22_ of mixtures of myofibrillar and sarcoplasmic protein solution.

		Drying Rate (min^−1^)	Drying Time (h)				Drying Rate × Drying Time
			0	3	6	9	
Peak 1 (T_2b_)	Relaxation time (ms)	0.0018	2.05 ± 0.08 ^b^	1.58 ± 0.59 ^b^	1.72 ± 0.75 ^b^	3.55 ± 0.83 ^a^	0.097
		0.00015	2.05 ± 0.08 ^b^	2.47 ± 0.19 ^b^	3.6 ± 0.55 ^a^	4.47 ± 0.93 ^a^	
	*p*-Value	0.000	0.002				
	Peak area	0.0018	97.55 ± 4.96 ^a^	98.8 ± 7.72 ^a^	84.14 ± 11.65 ^ab^	69.47 ± 10.78 ^b^	0.044
		0.00015	97.55 ± 4.96 ^a^	84.04 ± 10.95 ^ab^	53.16 ± 8.52 ^c^	46.79 ± 8.50 ^c^	
	*p*-Value	0.000	0.000				
	Ratio (%)	0.0018	1.06 ± 0.07 ^a^	0.97 ± 0.03 ^ab^	0.86 ± 0.17 ^bc^	0.77 ± 0.08 ^cd^	0.185
		0.00015	1.06 ± 0.07 ^a^	0.92 ± 0.06 ^abc^	0.65 ± 0.07 ^de^	0.58 ± 0.11 ^e^	
	*p*-Value	0.012	0.000				
Peak 2 (T_2b_)	Relaxation time (ms)	0.0018	14.6 ± 3.47 ^abc^	11.63 ± 0.46 ^bc^	14.42 ± 2.14 ^abc^	16.18 ± 2.26 ^ab^	0.977
		0.00015	14.6 ± 3.47 ^abc^	11.3 ± 2.51 ^c^	15 ± 0.61 ^abc^	16.91 ± 1.96 ^a^	
	*p*-Value	0.801	0.014				
	Peak area	0.0018	118.47 ± 19.78 ^a^	92.22 ± 5.31 ^bc^	98.81 ± 3.68 ^abc^	106.94 ± 5.72 ^ab^	0.652
		0.00015	118.47 ± 19.78 ^a^	79.81 ± 11.69 ^c^	82.49 ± 16.55 ^c^	90.66 ± 4.46 ^bc^	
	*p*-Value	0.045	0.002				
	Ratio (%)	0.0018	1.27 ± 0.22 ^a^	0.91 ± 0.03 ^cd^	1.12 ± 0.10 ^abc^	1.19 ± 0.02 ^ab^	0.856
		0.00015	1.27 ± 0.22 ^a^	0.87 ± 0.10 ^d^	0.99 ± 0.10 ^bcd^	1.12 ± 0.08 ^abc^	
	*p*-Value	0.290	0.001				
Peak 3 (T_21_)	Relaxation time (ms)	0.0018	200.66 ± 16.44 ^b^	219.64 ± 0.00 ^a^	200.33 ± 7.93 ^b^	191.16 ± 0.00 ^b^	0.967
		0.00015	200.66 ± 16.44 ^b^	219.64 ± 0.00 ^a^	200.33 ± 7.93 ^b^	195.75 ± 7.93 ^b^	
	*p*-Value	0.773	0.001				
	Peak area	0.0018	9015.22 ± 152.82 ^ab^	9964.49 ± 612.85 ^a^	8664.57 ± 1058.48 ^b^	8840.4 ± 561.90 ^ab^	0.575
		0.00015	9015.22 ± 152.82 ^ab^	9058.67 ± 475.01 ^ab^	8123.34 ± 1032.64 ^b^	7938.88 ± 226.08 ^b^	
	*p*-Value	0.037	0.019				
	Ratio (%)	0.0018	97.76 ± 0.36 ^bc^	98.02 ± 0.18 ^abc^	97.71 ± 0.46 ^bc^	98.05 ± 0.08 ^abc^	0.268
		0.00015	97.76 ± 0.36 ^bc^	98.45 ± 0.31 ^a^	98.36 ± 0.15 ^a^	98.28 ± 0.14 ^ab^	
	*p*-Value	0.012	0.049				

Only the different lowercase letters in each row indicate significant differences (*p* < 0.05).

## Data Availability

The original contributions presented in the study are included in the article, further inquiries can be directed to the corresponding author.
